# Polycyclic aromatic hydrocarbon mitigation in beef and camel steaks using plant juice and waste marinades

**DOI:** 10.1038/s41538-025-00501-z

**Published:** 2025-07-18

**Authors:** Doaa Nowar, Shimaa Edris, Islam Sabeq

**Affiliations:** Department of Food Hygiene and Control, Faculty of Veterinary Medicine, Benha University, Toukh, Qalyubia Egypt

**Keywords:** Risk factors, Chemical safety, Science, technology and society

## Abstract

This study assessed the consequence of milk-based marinades with plant/fruit juice or wastes on polycyclic aromatic hydrocarbons (PAHs) production in charcoal-grilled beef- vs. camel-*longissimus lumborum* steaks (*BLLS* vs. *CLLS*). Eight *BLLS* or *CLLS* batches were marinated in milk alone (control) or milk plus either 10% beetroot juice (*BTJM*), 1% dragon fruit peel extract (*DFPEM*), or 10% pomegranate-peel powder (*PGPPM*). Wrapping *BLLS* and *CLLS* in aluminium foil significantly reduced benzo[a]pyrene, and PAH4 levels below the maximum permissible values. All marinade durations and types effectively decreased PAH12, benzo[a]pyrene, and PAH4 levels in *CLLS* compared to *BLLS* (*P* < 0.05). Long-term marination greatly reduced PAH12, benzo[a]pyrene, and PAH4 levels compared to short-term (*P* < 0.05). Waste-based marinades and *CLLS* were related to greater PAH levels than other marinades and *BLLS*, respectively (*P* < 0.05). Finally, high-moisture, low-cellulose, and/or low-pectin acidic marinades, plus meat wrapping, are potentially affordable and efficient PAH reduction strategies prior to grilling.

## Introduction

The goal of cooking is to provide bacteriologically safe food with the best possible sensory qualities and the least amount of potentially hazardous ingredients. However, studies have shown that high cooking and food processing temperatures can generate a range of genotoxic chemicals, also referred to as cooking toxicants. In 2022, there were 19,976,499 cases of all malignancies in both sexes, with an anticipated 1,185,216 cases occurring in Africa (5.9%)^1^.

In the 1960s and the 1970s, concerns associated with polycyclic aromatic hydrocarbons (PAHs) in food started to evolve, with two groups of dietary toxicants that induce cancers receiving special attention: PAHs and N-nitroso compounds^2^. The PAH class consists of ~10,000 distinct chemical compounds that are nonpolar molecules with lipophilic, semi-volatile, and persistent properties, differentiated by their structure of hydrogen and carbon atoms^3^. There is still rising worry regarding the effects of these chemicals on human health^2^. Food practices, cooking methods, and the number of benzene rings affect an individual’s exposure. PAHs with two to three or four benzene rings are categorized as light or low-molecular-weight (LMW) PAHs, whereas PAHs with at least four or five rings in their structure are classified as heavy or high-molecular-weight PAHs (HMW)^4^. The most important and dangerous PAHs were evaluated and categorized by concerned organizations. The US Environmental Protection Agency (US EPA) initially published a list of 16 PAHs, and then the European Union Scientific Committee on Food (SCF) identified 15 heavy PAHs as much more genotoxic, mutagenic, and carcinogenic and recommended for monitoring in food. The Joint FAO/WHO Expert Committee on Food Additives reduced this number to 13. However, the EFSA Panel on Contaminants in the Food Chain (CONTAM) and the 2005 European Commission Recommendation found that B[a]P alone is not a suitable indicator of other PAHs in food products^5,6^. The greatest indicator of the presence of PAHs in food has now been determined to be the overall amount of four heavy PAHs (benzo[a]pyrene, chrysene, benzo[b]fluoranthene, and benzo[a]anthracene)^7^. Polyarenes were further divided into four categories of carcinogens by the International Agency for Research on Cancer (IARC): group 1 was defined as human carcinogens, group 2A as probable carcinogens, group 2B as possible carcinogens, group 3 as not classifiable as human carcinogens, and group 4 as probably not carcinogenic to humans^8^.

Controlling the numerous factors that contribute to the generation of PAHs is a strategy used to reduce their production. The primary factor is the smoke produced by the incomplete combustion of fuels, such as gas and charcoal, during cooking, which releases PAHs onto the surface of the food material. LMW PAHs are frequently formed when burning below 500, 200, and 300 °C^9^. Temperature and length of exposure also influence the production of PAHs; higher temperatures and longer exposure times result in the generation of HMW PAHs rather than LMW PAHs and hydrocarbon radicals^4,9,10^. Greater levels of HMW PAHs in smoked food products than in grilled foods are attributed to exposure factors, with grilling requiring less time than smoking^11^. A higher fat content in food promotes the development of PAHs, especially when exposed to flames. Along with fat content, processing time and temperature, cooking style, and distance from the heat source, the type of meat is one of the most important factors influencing PAH contamination in grilled foods^12^. Lipid oxidation is another important component that may contribute to the LMW PAHs generation at lower temperatures during extended storage. Research indicates that beef patties refrigerated for 9 days before grilling contained significantly higher levels of PAH8^13^. Food combustion, followed by the release of free radicals and their recombination, was assumed to be the cause of PAHs in food^14,15^. Higher PAH values were associated with foods with lower scavenging capacities. Previous investigations found that the production of PAHs might be mediated by free radical chains^13^. Therefore, reducing these free radicals by adding antioxidants or radical scavengers, particularly during the pre-treatment phase, is a likely way to reduce the production or buildup of PAHs in food products^15,16^. Antioxidants and spices have been shown to reduce PAH production^13,17^.

Compared to other foods, the generation of carcinogenic and mutagenic compounds, such as PAHs, in grilled and/or barbecued meat products poses serious risks; hence, research into alternative remedies is necessary^18^. Food manufacturing is a comprehensive process, and every stage, from pretreatments and ingredients to processing settings and formulations to food packaging and storage conditions, may have an impact on the amount of PAHs in the finished products^12^. A thorough examination of the factors that contribute to the production of PAHs and methods for reducing them along the whole food product manufacturing chain would be a useful resource for food scientists and producers^12^. Innovative methods and potential strategies for lowering PAH levels in food have recently been developed. These include PAH adsorption by lactic acid bacteria (LAB), cellulosic aerogel, PAH degradation by ozonation and ultraviolet (UV) irradiation, and PAH reduction by using fuel catalysts and edible coatings^12,19,20^.

Research is still required to develop affordable mitigation techniques for carcinogenic and mutagenic PAHs produced during meat grilling. Setting the temperature for a predetermined amount of time and incorporating functional smoking equipment with regulated smoke levels are important mitigation elements in the smoking process^18^. However, in barbecuing or traditional food grilling conditions, the temperature cannot be maintained for a certain time. Additionally, using appropriate smoking equipment with regulated smoke levels would be costly. Moreover, most previously mentioned innovative techniques, except for coating, are not simply applied at the home level. Consequently, the current study aimed to investigate field-affordable mitigation strategies utilizing milk-based marinades, integrating plant juice or fruit waste, to limit the generation of hazardous PAHs in grilled meat. The impact of the marination period and meat species (beef or camel) on PAH generation during grilling was also investigated.

## Results

### Marination impact on physicochemical attributes of beef and camel steaks over varying durations

The pH values of the various marinades applied to steaks of beef and camel over varying marination times are shown in Table 1. Compared to unmarinated meat, *PGPPM* exhibited the lowest acidic pH across all marination durations, followed by *DFPEM*, *BTJM*, and control milk.

**Table 1:** The pH of various marinades applied on beef and camel *longissimus lumborum* steaks across different marination durations (1 h vs. 4 days)

	Non-marinated		Marinated	
	1-h	4-days	1-h	4-days
*Beef*	6.75^cd^	6.25^g^		
*Camel*	7.43^a^	6.51^f^		
*Milk*			6.92^c^	6.92^c^
*BTJM*			7.01^b^	7^b^
*DFPEMM*			6.84^d^	6.79^e^
*PGPPM*			5.03^i^	5.94^h^
SEM	0.054	0.038	0.028	0.007
*P* value	<0.001	0.001	< 0.001	<0.001

*BTJM* beetroot juice extract marinade, *DFPEM* dragon fruit peel extract marinade, *PGPPM* pomegranate peel powder marinade, *LS* Longissimus species, *MRT* marinade type, *MD* marination duration, *SEM* standard error mean.

Different small letters within the row and/or column show significant differences between treatments and/or marination time (*p* < 0.05).

Table 2 shows the effect of different marinade types on the water-holding capacity (WHC), thawing loss, and cooking loss of *BLLS* and *CLLS* after varying marination times. The *LLS* WHC was not affected by any of the studied variables, according to the general linear model (GLM) (*P* > 0.05). However, only *LLS* species displayed a significant difference; *CLLS* had a numerically higher WHC than *BLLS* across both short- and long-term marination (*P* > 0.05), especially during the long-term DFPEM marination (*P* < 0.05).

**Table 2:** Influence of various marinades on the water-holding capacity, thawing loss, and cooking loss of beef- and camel-*longissimus lumborum* steaks across different marination durations (1 h vs. 4 days)

Marinades	Marination durations (MD)							
	Short-term (1 h)					Long-term (4 days)		
	Beef	Camel	SEM	*P* values	Beef	Camel	SEM	*P* values
Water holding capacity (WHC)								
Non-marinated	94.67 ^A^	86.88^AB^	2.107	0.070	81.71^B^	76.87 ^C^	0.684	0.002
Control	81.66	91.19	3.797	0.158	85.26	88.56	3.592	0.534
* BTJM*	90.1	91.98	3.278	0.533	91.6	93.69	1.368	0.4
* DFPEM*	80	91.24	5.582	0.327	83.49^B^	91.67 ^A^	1.649	0.01
* PGPPM*	87.8	89.59	2.096	0.541	86.7	91.17	2.178	0.323
SEM	3.612	3.764		2.205	2.205	2.189		
* P* values	0.603	0.945		0.229	0.229	0.89		
Thawing loss (ThL)								
Non-marinated	2.28^C^	11.51^AB^	1.073	0.038	7.34 ^AB^	14.07^A^	1.286	0.062
Control	7.87	19.74	3.944	0.188	10.69	17.75^b^	2.819	0.378
* BTJM*	9	16.63	3.329	0.341	11.59	18.85^b^	2.256	0.13
* DFPEM*	11.59	16.68	4.904	0.733	9.98	17.42^b^	2.2	0.063
* PGPPM*	17.20^AB^	14.73^BC^	2.288	0.72	15.06^AB^	28.77^aA^	5.682	0.38
SEM	2.089	2.409			2.179	1.873		
* P* values	0.484	0.947			0.91	0.02		
Cooking loss (CoL)								
Control	39.92^BC^	51.34^B^	3.183	0.048	52.44^AB^	62.61^A^	4.825	0.179
* BTJM*	46.07	49.32	8.146	0.796	53.43	46.03	3.732	0.36
* DFPEM*	47.85	52.15	4.857	0.562	52.91	53.62	5.439	0.945
* PGPPM*	48.53	55.44	2.806	0.164	53.06	47.88	2.934	0.392
SEM	4.795	4.701				2.295	6.171	
* P* values	0.6	0.867				0.993	0.855	
General linear model (GLM)			*WHC*		*ThL*		*CoL*	
SEM			1.079		0.978		0.81	
*P* values	*Longissimus* species (LS)		0.026		0.529		0.001	
	Marinade type (MRT)		0.307		0.276		0.082	
	Marination duration (MD)		0.627		0.514		0.102	
	LS * MRT		0.594		0.882		0.001	
	LS * MD		0.715		0.655		0.008	
	MRT * MD		0.99		0.408		0.009	
	LS * MRT * MD		0.894		0.237		0.745	

*BTJM* beetroot juice extract marinade, *DFPEM* dragon fruit peel extract marinade, *PGPPM* pomegranate peel powder marinade, *LS* Longissimus species, *MRT* marinade type, *MD* marination duration, *SEM* standard error mean.

Different small letters within the column show significant differences between treatments (*p* < 0.05), while different capital letters within the row indicate significant changes between species and marination time.

The GLM revealed that none of the variables under consideration influenced *LLS* thawing loss (*P* > 0.05). However, during both short- and long-term marination, *CLLS* exhibited numerically higher thawing losses than *BLLS* (*P* > 0.05). Furthermore, it was noticed that PGPPM was related to the largest cooking loss in both *LLS* species and most marination periods, particularly in the long-term marinated *CLLS*.

The cooking loss of charcoal-grilled *LLS* was statistically influenced by *LLS* species and their direct interactions with marinade type, marination duration, and marinade type and marination-duration interactions (*P* < 0.05). The GLM revealed that the control- and *PGPPM*-marinated *LLS* of either species resulted in the greatest cooking loss. Also, short-term marinated *BLLS* had a little lower cooking loss than *CLLS*, whereas longer-term marinated *CLLS*, excluding control, had a numerically lower cooking loss than *BLLS* (*P* > 0.05).

### The consequences of marination types and durations on the total estimated polycyclic aromatic hydrocarbons (PAH12) in beef and camel steaks

Table 3 shows the effects of different marinade types on the total estimated PAH12 in beef and camel flesh after differing marinating times. The *LLS* species, marinade type, marination duration, and their direct interactions (*P* > 0.05), all statistically significantly impacted the levels of PAH12 in charcoal-grilled *LLS* (*P* < 0.05). At all marination durations, short or long, all marinade types had no effect on total estimated PAH12 in *BLLS* as compared to the control marinade. In *CLLS* steaks, all marinade types significantly lowered levels, with the exception of *PGPPM*, which remained higher after long-term marination. In both species, long-term marination significantly decreased PAH12 compared to short-term marination. Higher levels of cellulose and/or pectin in fruit waste-derived marinades, *DFPEM*, and *PGPPM* relative to control marinade and *BTJM* were probably associated with increased levels of PAH12 in most *LLS* species, especially *BLLS*. Across all marination durations, *BLLS* was consistently associated with lower levels of PAH12, benzo[a]pyrene, and PAH4 than *CLLS* (*P* < 0.05).

**Table 3:** The impact of various marinades on the sum of total estimated polycyclic aromatic hydrocarbons (PAH12, ng/kg) in beef and camel *longissimus lumborum* steaks at varied marination durations (1 h versus 4 days)

Marinades	Marination durations (MD)							
	Short-term (1 h)				Long-term (4 days)			
	Beef	Camel	SEM	*P* values	Beef	Camel	SEM	*P* values
Control	255.03^bC^	1488.52 ^aA^	141.2	0.010	223.67^bC^	562.72^bB^	27.164	0.001
* BTJM*	315.96^abC^	643.02^bA^	25.69	0.001	207.24^bD^	303.17^cB^	12.280	0.007
* DFPEM*	352.87^aAB^	506.36^bA^	56.58	0.203	328.63^aB^	159.12^dC^	10.981	0.001
* PGPPM*	309.36^ab^	794.68^b^	43.42	0.001	357^a^	686.93^a^	37.706	0.003
SEM	19.807	113.656		23.495	23.495	20.570		
* P* values	0.072	0.006		0.006	0.006	<0.001		
General linear model (GLM)								
	SEM			24.749				
*P* values	*Longissimus* species (LS)			0.000				
	Marinade type (MRT)			0.005				
	Marination duration (MD)			0.000				
	LS * MRT			0.001				
	LS * MD			0.004				
	MRT * MD			0.114				
	LS * MRT * MD			0.208				

*BTJM* beetroot juice extract marinade, *DFPEM* dragon fruit peel extract marinade, *PGPPM* pomegranate peel powder marinade, *LS* Longissimus species, *MRT* marinade type, *MD* marination duration, *SEM* standard error mean.

Different small letters within the column show significant differences between treatments (*p* < 0.05), while different capital letters within the row indicate significant changes between species and marination time.

### The consequences of marination types and durations on the levels of group 1A carcinogenic polycyclic aromatic hydrocarbon index in beef and camel steaks

The consequences of different marinade types on the levels of group 1A carcinogenic PAH (benzo[a]pyrene) in *BLLS* and *CLLS* over varying marinating times are shown in Table 4. All evaluated charcoal-grilled *BLLS* and *CLLS* had benzo[a]pyrene levels below the 2 μg/kg threshold set by the CONTAM Panel of the European Food Safety Authority (EFSA)^21^. The *LLS* species, marinade-type, marination duration, and their interactions—except for the marinade type and marination duration interaction (*P* > 0.05)—all statistically significantly impacted the levels of benzo[a]pyrene in charcoal-grilled *LLS* (*P* < 0.05). Compared to *LLS* marinated with *DFPEM* and *PGPPM*, charcoal-grilled *BLLS* marinated for short-term with either milk or *BTJM* displayed lower levels of benzo[a]pyrene (*P* < 0.05). Benzo[a]pyrene levels were lower in *CLLS* shortly marinated for 1-h in *BTJM* or *DFPEM* than in *LLS* marinated in milk or PGPPM (*P* < 0.05). Compared to corresponding *BLLS*, charcoal-grilled *CLLS* shortly marinated (1 h) with any marinade, except for *DFPEM*, showed significantly higher levels of benzo[a]pyrene (*P* < 0.05). After long-term marination at 4 °C, *DFPE* marinated *BLLS* had statistically significantly higher amounts of benzo[a]pyrene compared to other groups, including control marinade (*P* < 0.05). The long-term marination of *CLLS* in *PGPPM* generated statistically the highest benzo[a]pyrene level, followed by the control, while the lowest levels were observed when marinated with *BTJM* and *DFPEM* (*P* < 0.05). Long-term (4-days) marinated charcoal-grilled *BLLS* in milk and *PGPPM* had lower benzo[a]pyrene levels than the corresponding *CLLS*; however, *CLLS* showed lower levels than *BLLS* in *BTJM* and *DFPEM* (*P* < 0.05). Both charcoal-grilled *BLLS* and *CLLS* had lower levels of benzo[a]pyrene after long-term marination compared to short-term. Nonetheless, the majority of marinades significantly reduced benzo[a]pyrene in *CLLS*, except *PGPPM*, which was the only marinade that caused a significant benzo[a]pyrene decrease in *BLLS* following long-term marination.

**Table 4:** Influence of various marinades on the level (ng/kg) of group 1A, classified carcinogenic, polycyclic aromatic hydrocarbons (benzo[a]pyrene) in beef and camel *longissimus lumborum* steaks across different marination durations (1 h vs. 4 days)

Marinades	Marination durations (MD)							
	Short-term (1 h)				Long-term (4 days)			
	Beef	Camel	SEM	*P* values	Beef	Camel	SEM	*P*
Control	9.54^bC^	51.21 ^aA^	3.72	0.002	5.34^bC^	26.94^bB^	2.56	0.008
* BTJM*	9.43^bBC^	29.38^bA^	2.25	0.003	3.49^bC^	3.26^cC^	0.49	0.785
* DFPEM*	22.64 ^aA^	20.84^bA^	4.64	0.797	10.9^aAB^	2.02^cC^	0.96	0.003
* PGPPM*	16.61^abB^	45.29 ^aA^	3.07	0.003	3.25^bC^	38.59 ^aA^	1.14	<0.001
SEM	3.127	3.713			0.784	1.793		
* P* values	0.066	0.002			0.001	<0.001		
General linear model (GLM)								
	SEM (GLM)			0.726				
*P* values (GLM)	*Longissimus* species (LS)			<0.001				
	Marinade type (MRT)			<0.001				
	Marination duration (MD)			<0.001				
	LS * MRT			<0.001				
	LS * MD			0.001				
	MRT * MD			0.476				
	LS * MRT * MD			0.007				

*BTJM* beetroot juice extract marinade, *DFPEM* dragon fruit peel extract marinade, *PGPPM* pomegranate peel powder marinade, *LS* Longissimus species, *MRT* marinade type, *MD* marination duration, *SEM* standard error mean.

Different small letters within the column show significant differences between treatments (*p* < 0.05), while different capital letters within the row indicate significant changes between species and marination time.

### The consequences of marination types and durations on the levels of group 2A probably carcinogenic polycyclic aromatic hydrocarbon index in beef and camel steaks

The impact of different marinades on the concentration of group 2A probably carcinogenic PAHs (dibenz[a,h]anthracene, D[a,h]A) in *BLLS* and *CLLS* across varying marination periods is shown in Table 5. The concentration of group 2A probably carcinogenic PAHs, dibenz[a,h]anthracene (D[a,h]A), in charcoal-grilled *LLS* was statistically significantly affected by *LLS* species, marinade-type, marination duration, and their interactions (*P* < 0.05). The findings of the short-term 1-h marination revealed that all marinades reduced D[a,h]A in *CLLS* when compared to the control; however, only the DFPEM demonstrated a significant reduction in *BLLS*. All applied marinades did not alter the D[a,h]A content of *BLLS* when marinated for 4 days (*P* > 0.05), but the long-term PGPP marination statistically significantly raised the D[a,h]A content of *CLLS* when compared to all other marinade types (*P* < 0.05). Furthermore, statistical analysis revealed that *CLLS* marinated under various short and/or long-term settings, especially control, DFPEM, and PGPPM (*P* < 0.05), had numerically higher D[a,h]A levels than *BLLS* (*P* > 0.05). Compared to benzo[a]pyrene findings, the long-term duration of most marinade types did not tend to lower D[a,h]A levels observed in short-term marinated *BLLS* or *CLLS* (*P* > 0.05), except for the control camel marinade (*P* < 0.05). However, long-term marination of *BLLS* with *DFPEM* and *CLLS* with *BTJM* and *PGPPM* raised D[a,h]A levels (*P* < 0.05).

**Table 5:** Influence of various marinades on the level (ng/kg) of group 2A, probably classified carcinogenic, polycyclic aromatic hydrocarbons in beef and camel *longissimus lumborum* steaks across different marination durations (1 h vs. 4 days)

Marinades	Marination durations (MD)							
	Short-term (1 h)				Long-term (4 days)			
	Beef	Camel	SEM	*P* values	Beef	Camel	SEM	*P* values
Control	0.89^aB^	697.62 ^aA^	116.93	0.041	1.19^B^	0.75^bB^	0.585	0.696
* BTJM*	0.7^aAB^	0.57^bC^	0.039	0.100	1.75 ^A^	1.48^bA^	0.343	0.601
* DFPEM*	0.06^bC^	0.67^bB^	0.091	0.009	1.41^AB^	0.40^bB^	0.243	0.094
* PGPPM*	0.83^aBC^	1.71^bB^	0.312	0.176	1.17^B^	6.1 ^aA^	0.597	0.008
SEM	0.068	58.620			0.304	0.580		
* P* values	<0.001	0.006			0.598	0.002		
General linear model (GLM)								
	SEM			14.614				
*P* values (GLM)	*Longissimus* species (LS)			0.005				
	Marinade type (MRT)			<0.001				
	Marination duration (MD)			0.006				
	LS * MRT			<0.001				
	LS * MD			0.006				
	MRT * MD			<0.001				
	LS * MRT * MD			<0.001				

*BTJM* beetroot juice extract marinade, *DFPEM* dragon fruit peel extract marinade, *PGPPM* pomegranate peel powder marinade, *LS* Longissimus species, *MRT* marinade type, *MD* marination duration, *SEM* standard error mean.

Different small letters within the column show significant differences between treatments (*p* < 0.05), while different capital letters within the row indicate significant changes between species and marination time.

### The consequences of marination types and durations on the levels of 2B-classified possibly carcinogenic polycyclic aromatic hydrocarbon index in beef and camel steaks

The impact of different marinades on the level of 2B-classified possibly carcinogenic PAHs, including B[a]A, Chr, B[b]F, and B(k)F, in *BLLS* and *CLLS* during varying marinating times is shown in Table 6. According to the International Agency for Research on Cancer (2010), there are five components that make up Group 2B PAHs: benzo[a]anthracene (B[a]A), chrysene (Chr), benzo[b]fluoranthene (B[b]F), benzo[k]fluoranthene (B(k)F), and indeno [1,2,3-cd] pyrene (I(c,d)P). However, I(c,d)P was not evaluated in the present study. Thus, the results may not be typical of 2B-classified, possibly carcinogenic, PAHs. The *LLS* species, marinade-type, and their interactions all statistically influenced the level of 2B-PAHs in charcoal-grilled *BLLS* and *CLLS* (*P* < 0.05). Nevertheless, 2B-PAHs in charcoal-grilled *BLLS* and *CLLS* were not substantially impacted by the marination duration or the interaction of marination duration with *LLS* species (*P* > 0.05). The short-term 1-h marination results showed that all marinades reduced 2B-PAHs in *CLLS* when compared to the control. In *BLLS*, only *BTJM* tended to exhibit a modest reduction (*P* > 0.05), while *DFPEM* and *PGPPM* increased 2B-PAHs levels (*P* < 0.05). All long-term marination resulted in a moderate or considerable increase in the 2B-PAH content of *BLLS* and *CLLS* steaks; however, at the long-term marination period, the *BTJM* considerably decreased the 2B-PAH content of *CLLS* (*P* < 0.05). Moreover, all *CLLS* marinated in different short- and long-term settings showed higher amounts of 2B-PAHs than *BLLS* (*P* < 0.05), except for the opposite correlation in short-term *PGPPM* and long-term *BTJM*. In contrast to benzo[a]pyrene findings, most marinade types at long-term marination increased 2B-PAHs levels in *BLLS* or *CLLS* compared to short marination duration (*P* < 0.05), except for the *DFPEM* in beef and *BTJM* and to a lesser degree control marinade in *CLLS*, which markedly reduced 2B-PAHs levels (*P* < 0.05).

**Table 6:** Influence of various marinades on the level (ng/kg) of 2B-classified, possibly carcinogenic, polycyclic aromatic hydrocarbons in beef and camel *longissimus lumborum* steaks across different marination durations (1 h vs. 4 days)

Marination durations (MD)								
	Short-term (1 h)				Long-term (4 days)			
Marinades	Beef	Camel	SEM	*P* values	Beef	Camel	SEM	*P* values
Control	15.11^cC^	359.58 ^aA^	18.717	0.001	30.02^bB^	211.62^bAB^	35.66	0.055
* BTJM*	12.34^cC^	240.3^bA^	7.152	<0.001	105.61^aB^	13.27^cC^	7.19	0.002
* DFPEM*	90.53^aC^	287.57^abAB^	15.647	0.001	33.09^bD^	373.3 ^aA^	19.4	0.001
* PGPPM*	40.75^bC^	18.44^cD^	2.970	0.011	122.59^aB^	310.74^abA^	15.50	0.001
MSE	4.035	18.21			9.02	29.85		
P values	<0.001	<0.001			<0.001	0.001		
General linear model (GLM)								
	SEM			5.759				
*P* values	*Longissimus* species (LS)			<0.001				
	Marinade type (MRT)			<0.001				
	Marination duration (MD)			0.151				
	LS * MRT			<0.001				
	LS * MD			0.169				
	MRT * MD			<0.001				
	LS * MRT * MD			<0.001				

*BTJM* beetroot juice extract marinade, *DFPEM* dragon fruit peel extract marinade, *PGPPM* pomegranate peel powder marinade, *LS* Longissimus species, *MRT* marinade type, *MD* marination duration, *SEM* standard error mean.

Different small letters within the column show significant differences between treatments (*p* < 0.05), while different capital letters within the row indicate significant changes between species and marination time.

### The consequences of marination types and durations on the concentration of the four polycyclic aromatic hydrocarbons index (PAH4) in beef and camel steaks

The impact of different marinades on the concentration of the PAH4, including benzo [a] anthracene (B[a]A), chrysene (Chr), benzo [b] fluoranthene (B[b]F), and benzo [a]pyrene (B[a]P), in *BLLS* and *CLLS* at varying marination durations is shown in Table 7. Compared to PAH4’s maximum permissible limit (MPL) of 12 μg/kg stipulated by European Union and adopted by the National Egyptian Food Safety Authority (NFSA), all investigated charcoal-grilled *BLLS* and *CLLS* exhibited lower acceptable levels. PAH4 levels in charcoal-grilled *LLS* were statistically significantly influenced by *LLS* species, marinade type, marination duration, and their interactions (*P* ≤ 0.05), except for the interaction of marination duration with either the marinade type or *LLS* species (*P* > 0.05). The PAH4 level in *BLLS* was considerably elevated by all short-term marinade types as compared to control marinade, except for *BTJM*, which statistically exhibited the lowest PAH4 level (*P* ≤ 0.05) after longer-term marinating. In line with benzo[a]pyrene results, marinating charcoal-grilled *CLLS* for 1 h or 4 days with *BTJM* and *DFPEM* reduced the amount of PAH4 in comparison to control and *PGPPM* (*P* ≤ 0.05). Additionally, charcoal-grilled *CLLS* marinated for a short- or long-term with any marinade type other than *DFPEM* were statistically significantly associated with greater levels of PAH4 (*P* ≤ 0.05) as compared to corresponding *BLLS*. All marinade types applied for a long-term duration significantly decreased PAH4 levels in both charcoal-grilled *BLLS* and *CLLS* when compared to short-term duration, which is consistent with benzo[a]pyrene findings. Most of the camel marination demonstrated a significant PAH4 decrease rate with long-term marination, except for *PGPPM*, which, along with *BTJM,* caused a strong PAH4 reduction influence in *BLLS*.

**Table 7:** Influence of various marinades on the level (ng/kg) of the four polycyclic aromatic hydrocarbons (PAH4) in beef and camel *longissimus lumborum* steaks across different marination durations (1 h vs. 4 days)

Marinades	Marination durations							
	Short-term (1 h)				Long-term (4 days)			
	Beef	Camel	SEM	*P* values	Beef	Camel	SEM	*P* values
Control	24.89^bC^	78.36 ^aA^	5.61	0.003	16.57^bcC^	47.54^bB^	3.57	0.004
* BTJM*	28.77^abAB^	52.53^abA^	7.45	0.089	7.34^cC^	20.88^cB^	1.25	0.002
* DFPEM*	52.66 ^aA^	38.55^bAB^	6.52	0.201	32.59^aAB^	14.14^cC^	2.18	0.005
* PGPPM*	49.81^aB^	76.44 ^aA^	5.56	0.042	23.53^abC^	70.72 ^aA^	2.59	<0.001
SEM	6.757	5.820			2.475	2.319		
* P* values	0.052	0.005			0.001	<0.001		
General linear model (GLM)								
SEM		1.267						
*P* values	Meat species (MS)	<0.001						
	Marinade type (MRT)	<0.001						
	Marination duration (MD)	<0.001						
	MT * MRT	<0.001						
	MT * MD	0.422						
	MRT * MD	0.520						
	MS * MRT * MD	0.037						

*BTJM* beetroot juice extract marinade, *DFPEM* dragon fruit peel extract marinade, *PGPPM* pomegranate peel powder marinade, *LS* Longissimus species, *MRT* marinade type, *MD* marination duration, *SEM* standard error mean.

Different small letters within the column show significant differences between treatments (*p* < 0.05), while different capital letters within the row indicate significant changes between species and marination time.

## Discussion

This study sought to evaluate field-affordable mitigation techniques that use milk-based marinades with plant juice or fruit waste to reduce the generation of hazardous PAHs in grilled meat. The effects of marinating time and meat type (beef or camel) on PAH production during grilling were also examined. Marination is a classic method of seasoning meat products by adding liquids infused with spices, flavorings, and other ingredients to enhance meat quality. Furthermore, the amount of PAHs in the finished product can be decreased by carefully selecting marinade ingredients^22–25^. Various ingredients, such as extracts, fruit juices, dairy products, and herbs, are typically used to make marinades.

The current investigation wrapped all marinated *BLLS* and *CLLS* in aluminum foil prior to charcoal-grilling, which could be one of the reasons that the levels of benzo[a]pyrene and PAH4 were lower than the maximum authorized limits despite the long charcoal-grilling duration. This finding can be explained by minimizing direct contact between meat and the charcoal grilling flame, which limits the pyrolysis of the organic components of meat, such as fat, proteins, and carbohydrates^26^. Furthermore, aluminum foil wrapping prevented fat dripping from leaking onto charcoal, preventing volatile PAHs from adhering to the surface of the meat placed over the heat source^27^. Studies have concluded that a significant decrease in PAHs was achieved by cutting off the contact of fat droplets with the heating source^11,27–29^. Unsaturated fatty acids have been shown to have a favorable association with the level of PAHs4, indicating their significant role in the development of PAHs^30^.

The type of meat and its fat content are two factors that determine the concentration of PAHs in cooked meat^31^. The current findings indicated that, under the same marination treatments and charcoal-grilling conditions, *BLLS* had significantly lower PAH12, benzo[a]pyrene, and PAH4 levels than *CLLS* after a short- or long-term duration of all marinades. Exceptionally, in *DFPEM*, *BLLS* had either similar or higher PAH12 levels than *CLLS* after short- and long-term durations, respectively. Similarly, *CLLS* marinated in various short- and/or long-term settings, particularly short-term control and DFPEM and long-term *PGPPM*, were linked with higher D[a,h]A levels than *BLLS*. Additionally, all *CLLS* were linked to greater 2B-PAHs levels than *BLLS* (*P* ≤ 0.05), except for lower levels observed in short-term *PGPPM* and long-term *BTJM* marinades (*P* ≤ 0.05). Compared to salmon, poultry, and pork, lean beef is known to produce fewer PAHs^13,17,32,33^. According to the species comparison, camels had higher levels of PAHs than beef did. Lean beef was linked with a higher fat content than camel^34^; hence, it was expected to contain more PAHs. Higher fat content in food promotes the development of PAHs, particularly when exposed to flames^11,27–29,35^. However, foil wrapping reduced the fat leakage-derived PAHs, indicating the involvement of other components.

Studies have linked camel meat to higher pH, lower WHC, and greater purge and cooking losses compared to beef. Camel meat has a higher inherent pH than beef, which may provide an alkaline environment. The current pH results revealed that *CLLS* had a higher pre-marination pH than *BLLS*; however, the pH decreased dramatically during marination. Compared to the pre-marination pH, the *PGPPM* had the lowest acidic pH across all marination durations, followed by *DFPEM*, *BTJM*, and control milk. Meat samples treated with an acidic marinade (including 1.2% lemon juice) reduced the quantity of PAHs in grilled beef by 70%^36^. Even though the current *PGPPM* has an acidic pH, it actually had more PAH12, benzo[a]pyrene, and PAH4 compared to the other marinades. The PAH levels in -marinated *LLS* were roughly comparable to those in *PGPP*-marinated *LLS*, which is probably attributable to the increased cellulose and pectin content in both marinades. The percentage of administered marinades (10 vs. 1%) may explain the variations in PAHs produced between the *PGPPM* and *DFPEM*. Conversely, lower pH and cellulose and/or pectin in *BTJM* may account for the lowest levels of PAHs, whereas greater pH associated with control milk may account for higher levels. Prior research has shown that adding basic amino acids, such as arginine and lysine, produces more PAHs than adding acidic amino acids. This is probably because the pH rises from the former, accelerating the Maillard process that creates PAHs and 1-amino-1-deoxy-2-ketosaccharide^37^. According to certain earlier studies, the percentage of purge loss in camel and mutton meat was twice as high as that in beef, and it rose over time^38^. Additionally, our most recent survey data in the study area showed that the average cooking loss for camels of various ages was 39.51%, while the average for beef was 32.63%^39^. Additionally, this suggestion was supported by the current slightly higher cooking loss of marinated *LLS* and the higher pre-marination and marination thawing losses of camels compared to beef. This higher cooking loss in camels led to less moisture and increased the organic component pyrolysis, accelerating the PAHs generation. Marination generally increases the WHC of meat, reduces cooking losses, and improves color and juiciness^24^. Current marination may have helped maintain the moisture content of camels’ *LLS* and reduced the rate of organic component pyrolysis, slowing PAH generation. This finding is consistent with multiple other research determined that water suppresses the formation of PAHs because it prevents incomplete combustion by acting as an oxygen source during heating^16,40^. Furthermore, it is critical to evaluate the contribution of aluminum foil wrapping in preserving the marinade coating *LLS* and reducing moisture loss, especially in camels.

Based on the similarity of the moderating consequences of marinades, the currently evaluated PAHs were categorized into two main groups: the first group involved PAH12, benzo[a]pyrene, and PAH4 levels, and the second group involved D[a,h]A and 2B classified PAHs. Previous studies have indicated that preprocessing beef, chicken, and pork meat with antioxidant-rich marinades and/or plant-derived extracts and/or juices, including green tea, cinnamon, onion, rosemary, and lemon, can minimize or mitigate the level of PAHs in the finished product^17,24,25,28,36,41^. One significant factor that may contribute to the production of LMW PAHs is lipid oxidation; diets with reduced scavenging capacity were linked to higher PAH levels. Free radical chains are involved in the formation of PAHs, especially LMW^13^. Consequently, lowering these free radicals using antioxidants or radical scavengers, especially in the pre-treatment stage, is probably a good method to decrease the synthesis or accumulation of PAHs in foodstuffs^15,16^. In this study, the detrimental effects of marinades on benzo[a]pyrene and PAH4 during long-term marination, and on D[a,h]A and 2B-PAHs after short-term marination, might be partially related to the antioxidant effects of marinades that adversely affect PAH synthesis. The PAH mitigating effect of marinades was shown to be significantly linked to their DPPH radical scavenging activity^17^. Betanins, saponins, polyphenols, and organic acids are beetroot phytochemicals that can withstand simulated gastrointestinal digestion^42^. The pomegranate peel, which makes up half of its weight and is often thrown away by the agricultural industry, can be a useful source of healthy compounds like flavonoids, anthocyanins, and tannins that give pomegranates their antioxidant and anti-radical properties. Flavonoids, particularly anthocyanins, contribute to the red color and have antibacterial and antioxidant qualities^43^. The peel extract of *H. polyrhizus* included 24 different components, accounting for 90.66% of the overall composition. Of these, triterpenoids made up 29.77% and steroids 16.46%. DFP has a high phenolic content, particularly gallic and ferulic acid (36–39 mgGAE/100 g), which provides excellent radical scavenging and reducing power^44^. Additionally, *DFP* contained 56.91% dietary fiber, which included a larger percentage of insoluble dietary fiber, along with 10.36% protein, 4.48% fat, and 2.34% ash^44^.

Natural antioxidants in marinades may quench or scavenge free radicals generated by oxidation and heat source interaction during grilling. Additionally, these antioxidant compounds may help shield the *LLS* oxidative sites or species, which could prevent the Maillard reaction and reduce the production and accumulation of PAHs^15^. Though antioxidants have a significant moderating effect on free radicals and reactive oxidative species, and hence on PAHs, various factors can interact to minimize PAH levels. Previous research has demonstrated that phenolic chemicals supply hydrogen to free radicals, transforming them into phenoxyl radicals. Additionally, phenolic compounds produce PAHs during pyrolysis, while cellulose and pectin serve as donors for the formation of PAHs^15^. Here, higher cellulose and/or pectin and their concentration in waste-derived marinades, DFPEM, and *PGPPM*, were directly related with higher PAH12, benzo[a]pyrene, and PAH4, notably in beef.

The marination duration displayed a notable impact on PAH levels, especially in camel meat rather than beef. All marinating periods, especially long-term ones, exhibited a significant moderating influence on the levels of PAH12, benzo[a]pyrene, and PAH4 in *CLLS*, except for *PGPPM*. In *BLLS*, the long-term marination time apparently had a stronger PAH-mitigating impact than the short-term one, especially with regard to *PGPPM* or *BTJM*. Nonetheless, a noteworthy but not conclusive observation was that, in comparison to longer-term marination, short-term marination displayed stronger mitigation on D[a,h]A and 2B-PAHs, either significantly as in *CLLS* or slightly as in the majority of treated *BLLS*. This is probably caused by the gradual deterioration of bioactive antioxidant components, particularly in marinades with a high peel powder percentage (*PGPPM*). This effect was previously noted with the bioactive antioxidant components of cinnamon powder, cinnamon aldehyde, and green tea powder, catechin^41^. Thus, it might be concluded that longer-term marination has an adverse impact on the sum of twelve estimated PAHs, benzo[a]pyrene and PAH4, yet correlated with higher D[a,h]A and 2B-PAHs in both meat species. Additionally, camel marination exhibited a larger reduction effect on PAHs than beef.

In conclusion, wrapping marinated *BLLS* and camel *CLLS* in aluminum foil considerably lowered benzo[a]pyrene and PAH4 levels below the maximum permissible values. All marinades in different short- and/or long-term settings, except for *PGPPM*, had a greater detrimental effect on the levels of PAH12, benzo[a]pyrene, and PAH4 in *CLLS* steaks than in *BLLS* steaks. Long-term marination (4 days) significantly lowered the estimated PAH12, benzo[a]pyrene, and PAH4 levels in both *LLS* species compared to short-term marination. However, long-term marination also resulted in higher amounts of D[a,h]A and 2B-PAHs in both types of meat. In comparison to control and *BTJM* marinades, higher amounts of cellulose and/or pectin in fruit waste-derived marinades, *DFPEM,* and *PGPPM* were linked to higher levels of PAHs in most *LLS* species or marinating periods. Regardless of marinating duration, *BLLS* were consistently associated with lower amounts of PAH12, benzo[a]pyrene, and PAH4 than *CLLS*. In conclusion, meat marinades with high moisture, low pH, and low cellulose and/or pectin, along with meat wrapping, have the potential to be a low-cost and effective PAH mitigation strategy prior to grilling. Species-specific physicochemical attributes of meat potentially impact PAHs formation during charcoal-grilling. Future research is also needed to compare and validate the effects of different meat species, marinades with varying cellulose and moisture concentrations, and other packaging materials on the generation of PAHs.

## Methods

### Experiment management and approval

The methods employed in this work were authorized by the Institutional Animal Care and Use Committee Research Ethics number (BUFVTM) of Benha University’s Faculty of Veterinary Medicine, with the number BUFVM 17-08-2024.

### Vegetable juice and fruit peel powder preparation

The control marinade was just pasteurized milk purchased from surrounding supermarket. The fresh beetroot (*Beta vulgaris*) (2 kg), red dragon fruit (*Hylocereus polyrhizus*) (2 kg), and pomegranate (*Punica granatum* L.) (2 kg) were acquired from the farm and retail markets in Benha, Egypt. Before the peel was removed, the entire fruit was cleaned and sprayed with alcohol. The pomegranate (*Punica granatum* L.) and red dragon fruit (*Hylocereus polyrhizus*) peels were dried for 24 h at −35 °C using freeze-drying or lyophilization (Christ alpha 1-2 LD Freeze-dryer, Osterode am Harz, Germany). To get the juice, the beetroot was cleaned, peeled, and the bulb mashed in a home cold press juicer machine (Tornado, Elaraby, Benha, Egypt). The mixture was then sieved. A 10% beetroot juice-marinade (*BTJM*) was made with 50 mL of *BTJ* combined with 450 mL of milk. To obtain a 10% *PGP* marinade, 50 g of pomegranate peel powder was blended with milk upto 500 mL. Ten grams of powdered *DF* peel were infused with 200 mL of distilled water, boiled at 100 °C for 60 min over a heated magnetic stirrer, and sieved to provide 5% *DF* extract (*DFPE*). To obtain 1% *DFPE*-marinade (*DFPEM*), 100 mL of 5% *DFPE* was combined with milk upto 500 mL.

### Sample preparation and marinade distribution

Fresh ribeye loin (*Musculus longissimus thoracis* et *lumborum*, *LLS*, 10-13th rib) from two young beef and two young camel carcasses were acquired from a nearby butcher shop on the day of slaughter and sent right away to the lab for analysis. From both sides of each carcass, *LLS* weighing 70 ± 5 g (about 3.5 × 6 cm^2^ and 1 cm thick) were aseptically produced. Subsequently, *LLS* of beef or camel were assigned at random to one of four milk-based marinades: milk alone (control), 10% handmade beetroot juice marinade (*BTJM*, V/V), 1% extracted dragon fruit peel marinade (*DFPEM*, V/V), or 10% pomegranate peel powder marinade (*PGPPM*, Wt/V). The allocation was carefully arranged to ensure that the two carcasses *LLS* steaks were included in all four treatments throughout the two marination durations (1 h and 4-days). The individual treatment included twelve steaks divided into two replicates, each with six pieces. Each replication received three steaks each storage-point for the 2 testing durations. After 1 h marination in the corresponding marinade, six samples of each species specified treatments were packaged into polypropylene (PP) zip-lock containers aerobically and stored in the freezer at −20 °C and thawed before charcoal-grilling. Each marinated sample was wrapped in tagged aluminum foil and placed in a random pattern on the stainless-steel grill with a wooden handle before the tough charcoal-grilling for 20 min, turning over every 2 min. Each grilled steak was diced, and 10 g of the chopped sample were used for PAH analysis.

### pH analysis

The non-marinated raw meat and marinade pH on the first marination day and after 4 days of marination were directly evaluated using pH-meter electrodes (Jenway 3510 pH-meter, Cole-Parmer, Staffordshire, United Kingdom). At room temperature, the pH meter was calibrated with three different pH levels (10, 4, and 7) and a temperature metal probe.

### Estimation of water holding capacity, thawing, and cooking losses

The simple and rapid filter paper press method was used to assess the WHC of *LLS*. This procedure entailed putting 0.2–0.4 g of sample on Whatman No. 1 filter paper and pressing it for 5 min with a weight of 5 kg. The amount of loose water divided by the sample’s initial weight, after the top plate has been removed, is the meat WHC^45^. To determine thawing loss, the *LLS* was initially weighed and stored at −21 °C for 1 week. The frozen *LLS* were then thawed at 5 °C for 24 h and the final weight was calculated. Thawing loss was calculated as a percentage of the difference between the initial and final weights^46^. Cooking loss was calculated as the difference in weight of *LLS* before and after charcoal grilling^46^.

### Polycyclic aromatic hydrocarbon (PAH) extraction

After the sample was thawed, a fraction of 10 g of each frozen sample was used to extract and clean up PAHs in charcoal-grilled samples. A grinder was used to homogenize each sample after it had been moved to a polypropylene (PP) container. A triplicate aliquot of the homogenized sample was then subjected to alkaline digestion to aid in tissue penetration and subsequent extraction, in which a 1 g aliquot of each sample was combined with 5 mL of a 2 M KOH solution in methanol and agitated on an oscillator shaker for 2 min. After that, 10 mL of *n*-hexane were added to the extracts, and they were sonicated for 5 min in a bath from JP Selecta (Barcelona, Spain) to undergo ultrasound-assisted extraction (UAE). After that, the unsaponifiable fat was separated from the organic layer by centrifugation at 5000 rpm (2150 × *g*) for 10 min at a low temperature (4 °C) on a JP Selecta rotator. After being evaporated under a nitrogen stream, the extract was redissolved in 25 ml of an aqueous solution that contained 4% acetonitrile^47^. The solid phase extraction (SPE) method used for cleanup the final extract. To condition the SPE columns, 1 mL of acetonitrile, methanol, and filtered water were added sequentially. The organic extract was kept in a 0.5 mL amber glass vial at −18 °C before analysis. Ultimately, the extracts were analyzed by injecting 1 μL aliquots into the GC–MS apparatus^47^.

### Gas chromatography–mass spectrometry (GC-MS) analysis

GC operating conditions: a Thermo Scientific TRACE GC UltraTM system (Thermo Fisher Scientific, Waltham, MA, USA) was used for the GC analysis. The GC was equipped with a Thermo TR-50MS 30 m, I.D. 0.25 mm, 0.25 μm film capillary column. GC conditions included splitless injection mode with a 5 mm injection port liner, injection port temperature of 270 °C, and flow rate of 1.2 mL/min. The split flow setting is set to “On” with a flow rate of 25 mL/min and a splitless time of 1 min. The *SSL* carrier method mode was set to constant flow with an initial value of “On”, a rate of 1.2 mL/min, and an initial time of 1 min. The gas saver flow was set at 15 mL/min, and the gas saver time was set to 3 min. The temperature of the transfer line was 270 °C, and the vacuum compensation was set to “On”. The oven temperature was set to 60 °C for 1 min, then programmed at 10 °C/min to 250 °C, then 20 °C/min to 280 °C.

Mass spectrometric conditions: a TSQ 8000 evo triple quadrupole mass spectrometer (Thermo Fisher Scientific, Waltham, MA, USA) was used for MS analysis. The mass spectrometer was tuned satisfactorily when the detector was adjusted to m/z 300 or below, the three FC 43 (calibration gas) ions (68, 219, and 502) were at least half the height of their respective windows, and the ions at 502 and 503 were resolved. The MS conditions for PAHs are listed below: the ionization mode was EI positive ion, the ion volume was close to EI, the emission current was 50 μA, and the ion source temperature was 250 °C. The scan type was Full scan in the m/z range 45–650 and SRM, with a scan width of 0.15 for SRM and a scan length of 0.2 s for full scan and 0.05 for SRM. Additionally, the peak widths were Q1, 0.7 Da; and Q3, 0.7 Da FWHM. Finally, the collision gas (Ar) pressure reached 0.5 mTorr.

For standard curve estimation, acetone was used to create a stock solution of each PAH at a concentration of 5 g/L, which was then kept at 4 °C. Fresh stock solutions were converted into working-strength solutions. The eluent, which was made daily in the lab, was acetonitrile with 100 μg/L of the internal standard triphenyl phosphate^47^.

### Statistical analysis

The data were analyzed with SPSS Version 22 (SPSS Inc., Chicago, IL, USA). The impact of meat species (beef and camel) and marination type on PAH levels in charcoal-grilled *LLS* was investigated using a two-way ANOVA test. While the muscle was considered random, the animal species and marination type and duration were considered fixed variables. To examine the impact of marinades on the production of PAHs in comparison to control milk, a one-way ANOVA was performed. The differences are shown by the superscript little letter in the columns. Non-marinated *LLS* WHC and thawing loss values were displayed in tables to easily compare the effect of marinades on these properties. However, because additional variables would be included, non-marinated *LLS* WHC and thawing loss values were not statistically included in one-way ANOVA and were not discussed. The results’ means and overall standard errors are displayed. The statistical model used Tukey’s b multiple comparison test. A significant difference was considered as a *P*-value < 0.05^48^.

## Supplementary information

Supplementary Table 1.

## Data Availability

All materials described in the manuscript, including all relevant raw data, were included as main and supplementary tables and figures. The datasets generated during and analyzed during the current study are available from the corresponding author upon reasonable request.
