# Supplementary material for: Polycyclic aromatic hydrocarbon mitigation in beef and camel steaks using plant juice and waste marinades
Source: NPJ Sci Food. 2025 Jul 18;9:141. doi: 10.1038/s41538-025-00501-z (PMC12274582; doi:10.1038/s41538-025-00501-z)
Supplement: Supplementary file 1 — Supplementary Table 1. [file 41538_2025_501_MOESM1_ESM.docx]

Supplementary Table 1. polycyclic aromatic hydrocarbons concentration in beef and camel *longissimus lumborum* steaks across different marination durations (one hour vs. four days) (ng/kg).

| PAH | Meat type | Marinades | | | | | | | | MSE | *P* values | | | | | | |
| --- | --- | --- | --- | --- | --- | --- | --- | --- | --- | --- | --- | --- | --- | --- | --- | --- | --- |
|  |  | Control | | BTJM | | DFPEM | | PGPPM | |  | MT | MR | ST | MT * MR | MT * ST | MR * ST | MT * MR * ST |
|  |  | 1 hr | 4 d | 1 hr | 4 d | 1 hr | 4 d | 1 hr | 4 d |  |  |  |  |  |  |  |  |
| AcN | Beef | 0.23 | 0.23 | 0.24 | 0.29 | 0.24 | 0.23 | 0.21 | 0.24 | 0.006 | 0.694 | 0.021 | 0.711 | 0.660 | 0.444 | 0.337 | 0.936 |
|  | Camel | 0.25 | 0.22 | 0.27 | 0.31 | 0.22 | 0.22 | 0.25 | 0.23 |  |  |  |  |  |  |  |  |
| Phen | Beef | 0.78 | 0.82 | 0.80 | 0.86 | 0.91 | 0.79 | 1.13 | 0.92 | 0.031 | 0.220 | 0.109 | 0.319 | 0.922 | 0.904 | 0.944 | 0.292 |
|  | Camel | 0.97 | 0.90 | 1.08 | 0.80 | 0.90 | 0.84 | 1.02 | 1.16 |  |  |  |  |  |  |  |  |
| ANT | Beef | 1.31 | 1.26 | 1.28 | 1.38 | 1.42 | 1.30 | 1.34 | 1.45 | 0.037 | <0.0001 | 0.015 | 0.846 | 0.042 | 0.759 | 0.024 | 0.130 |
|  | Camel | 2.03 | 1.38 | 1.42 | 1.47 | 1.50 | 1.45 | 1.78 | 2.28 |  |  |  |  |  |  |  |  |
| Pyrene | Beef | 0.36 | 0.34 | 0.37 | 0.33 | 0.39 | 0.41 | 0.39 | 0.39 | 0.016 | 0.867 | 0.783 | 0.842 | 0.595 | 0.584 | 0.985 | 0.815 |
|  | Camel | 0.40 | 0.41 | 0.32 | 0.40 | 0.40 | 0.37 | 0.29 | 0.33 |  |  |  |  |  |  |  |  |
| Fluorene | Beef | 26.94 | 26.96 | 26.91 | 26.92 | 26.96 | 26.97 | 26.91 | 26.94 | 0.538 | 0.994 | 1.000 | 0.994 | 1.000 | 0.981 | 1.000 | 1.000 |
|  | Camel | 26.96 | 26.95 | 26.97 | 26.92 | 26.96 | 26.93 | 26.90 | 26.85 |  |  |  |  |  |  |  |  |
| B[a]A | Beef | 1.96 | 1.93 | 1.93 | 1.92 | 1.94 | 1.92 | 1.92 | 1.95 | 0.060 | 0.891 | 0.996 | 0.909 | 1.000 | 0.860 | 1.000 | 2.000 |
|  | Camel | 1.97 | 2.00 | 1.92 | 1.95 | 1.92 | 1.96 | 1.92 | 1.97 |  |  |  |  |  |  |  |  |
| Chr | Beef | 7.70 | 6.81 | 11.15 | 0.19 | 11.34 | 14.42 | 21.01 | 15.76 | 0.634 | <0.0001 | <0.0001 | 0.003 | <0.0001 | 0.621 | 0.188 | 0.086 |
|  | Camel | 24.11 | 17.90 | 20.56 | 13.58 | 14.32 | 7.15 | 28.49 | 29.77 |  |  |  |  |  |  |  |  |
| B[k]F | Beef | -0.23 | 1.11 | 71.19 | 36.89 | 0.00 | 83.92 | -0.12 | 102.31 | 5.486 | <0.0001 | <0.0001 | <0.0001 | <0.0001 | <0.0001 | <0.0001 | 0.067 |
|  | Camel | 332.43 | 219.70 | 264.41 | 0.81 | 193.92 | 1.15 | 342.15 | 278.61 |  |  |  |  |  |  |  |  |
| B[b]F | Beef | 5.68 | 2.48 | 6.26 | 1.74 | 16.74 | 5.34 | 10.28 | 2.57 | 0.292 | <0.0001 | <0.0001 | <0.0001 | 0.003 | <0.0001 | 0.129 | 0.034 |
|  | Camel | 1.06 | 0.69 | 0.68 | 2.10 | 1.47 | 3.01 | 0.74 | 0.38 |  |  |  |  |  |  |  |  |
| B[a]P | Beef | 9.54 | 5.34 | 9.43 | 3.49 | 22.64 | 10.90 | 16.61 | 3.25 | 0.726 | <0.0001 | <0.0001 | <0.0001 | <0.0001 | 0.001 | 0.476 | 0.007 |
|  | Camel | 51.21 | 26.94 | 29.38 | 3.26 | 20.84 | 2.02 | 45.29 | 38.59 |  |  |  |  |  |  |  |  |
| B[ghi]P | Beef | 199.85 | 175.17 | 185.69 | 131.47 | 270.24 | 181.01 | 228.85 | 200.04 | 5.123 | <0.0001 | <0.0001 | <0.0001 | <0.0001 | 0.205 | 0.079 | 0.661 |
|  | Camel | 349.49 | 264.86 | 295.45 | 250.11 | 243.24 | 113.62 | 344.15 | 300.65 |  |  |  |  |  |  |  |  |
| D[ah]A | Beef | 0.89 | 1.19 | 0.70 | 1.75 | 0.06 | 1.41 | 0.83 | 1.17 | 14.614 | 0.005 | <0.0001 | 0.006 | <0.0001 | 0.006 | <0.0001 | <0.0001 |
|  | Camel | 697.62 | 0.75 | 0.57 | 1.48 | 0.67 | 0.40 | 1.71 | 6.10 |  |  |  |  |  |  |  |  |
| 1Beetroot juice (BTJM), dragon fruit peel extract (DFPEM), pomegranate peel powder (PGPPM), Meat species (MS), Marinade Type (MRT), Marination Duration (MD), General linear model (GLM), standard error mean (SEM), Acenaphthylene (AcN), Phenanthrene (Phen), fluorene (F), anthracene (ANT), benzo[a]pyrene (B[a]P), benzo[a]anthracene (B[a]A), benzo[b]fluoranthene (B[b]F), benzo[k]fluoranthene (B[k]F), benzo[g,h,i]perylene (B[ghi]P), chrysene (Chr), dibenzo[a,h]anthracene (D[ah]A), indeno[cd]pyrene (I[cd]P) | | | | | | | | | | | | | | | | | |
